# For Better Orchiopexy, Processus Vaginalis Should Be Dissected and a High Ligation Should Be Performed

**DOI:** 10.5041/RMMJ.10247

**Published:** 2016-07-28

**Authors:** Kaan Sonmez, Ramazan Karabulut, Zafer Turkyilmaz, Cem Kaya, Yildiz Pehlivan, A. Can Basaklar

**Affiliations:** Department of Pediatric Surgery, Medical Faculty of Gazi University, Ankara, Turkey

**Keywords:** Hernia, high ligation, processus vaginalis, undescended testis

## Abstract

**Objective:**

Data on the prevalence of patent processus vaginalis (PPV) and hernia in patients with cryptorchidism are controversial. While some pediatric surgeons do not dissect the processus vaginalis (PV), most prefer to do so to prevent hernia formation and to achieve an effective orchiopexy outcome. This study was performed to evaluate the importance of dissection and high ligation of the PV during treatment of undescended testis (UT).

**Methods:**

The clinical findings and surgical procedures of 55 patients with UT were retrospectively investigated.

**Results:**

The mean patient age was 2.5 (range 1.0–12.0) years. Non-palpable testis (NPT) was located on the right and left side in 39 and 16 patients, respectively. Ultrasonography revealed no testis in 10 patients and an atrophic testis in 7 patients. Seven patients had a parent with an inguinal hernia, and the silk sign or a PPV was detected during inguinoscrotal examination in 22 patients. Undescended testis repair was performed by an inguinal approach in all patients. The inguinal canal was opened in all patients; 42 patients had a wider-than-normal internal ring (>2.5 cm), and the posterior wall of the inguinal canal was consequently weakened. Two-stage orchiopexy was performed in 2 patients, and 15 underwent the Prentiss maneuver. In the remaining patients, the dissection was easily done, and the orchiopexy was performed without any difficulty. Scrotal edema and wound infection occurred in five and two patients, respectively. One patient presented with an atrophic testis, and three had recurrent UT. Inguinal hernia was not observed in any of the patients during the study period, and all procedures were performed on an outpatient basis.

**Conclusion:**

High ligation of the PV is an effective method for successful orchiopexy and prevention of inguinal hernia in patients with NPT and UT.

## INTRODUCTION

The incidence of undescended testis (UT) in pediatric patients >1 year of age is 2.2%. Approximately 20% of these patients have a non-palpable testis (NPT). In about half of patients with NPT, one testis is located in the abdominal cavity; the remaining testis is often atrophic, either secondary to antenatal torsion *in utero* or agenesis.^1^,^2^ Data on the prevalence of patent processus vaginalis (PPV) and hernia in patients with cryptorchidism are controversial.^3^ While some pediatric surgeons do not dissect the processus vaginalis (PV), most prefer to do so to prevent hernia formation and to achieve an effective orchiopexy outcome.^4^,^5^ The aim of the present study was to evaluate the importance of dissection of the PV from the spermatic cord and vessels during correction procedures for UT at the internal ring level or in an intra-abdominal location.

## METHODS

In total, 1,300 patients with UT and 55 with intra-abdominal NPT underwent operations in our clinic from 1998 to 2013. This retrospective study reviewed the records of the 55 children who underwent inguinal orchiopexy for treatment of intra-abdominal NPT. The age, period from diagnosis to operation date, side of UT, clinical findings, and surgical procedures were retrospectively investigated.

## RESULTS

The mean age of the patients was 2.5 (range 1.0–12.0) years. The NPT was located on the right side in 39 patients and on the left side in 16 patients. Preoperative physical examination revealed a palpable mobile mass at the internal ring level that was thought to be the UT in 41 (75.5%) patients; the testes were not palpable in the remaining patients. Scrotal and abdominal ultrasonography was performed in all patients. Ultrasonography revealed no testis in 10 patients and an atrophic testis in 7 patients. Seven patients had a parent with an inguinal hernia. The silk sign or a PPV was detected during inguinoscrotal examination in 22 patients. No patients displayed inguinal pain or signs of incarceration such as nausea, vomiting, or erythema of the scrotum or inguinal area.

Undescended testis repair was performed by an inguinal approach in all patients. The inguinal canal was opened in all patients; in 42 patients, the internal ring was wider than normal (>2.5 cm), and the posterior wall of the inguinal canal was consequently weakened. Furthermore, the PV was very delicate and fragile and the testes seemed to be in the PV during testicular retraction. The PPV was oriented as in inguinal hernia repair, allowing for free movement of intra-abdominal fluid. The PV was dissected from the pampiniform plexus and vas deferens during the surgical descent of the testis. Specifically, the PV was dissected to the level of the preperitoneal fat tissue, and high ligation was performed. During this procedure, the PV was very wide and thin in 15 patients and was opened. On the other hand, a purse string could be performed in 22 patients, and high ligation was performed. In patients in whom the testis could be moved into the widened PV sac and in cases involving extreme tension, PV dissection began from the region corresponding to the mid-distance between the internal ring and the testis. The aim of this maneuver was circumferentially to dissect the PV from a narrower diameter and allow for easier dissection of the PV, even in cases involving perforation of the PV.

Two patients required two-stage orchiopexy, and the Prentiss maneuver was performed in 15 patients, effectively reducing the testis to its anatomical position. In the remaining patients, the dissection was easily performed, and the testes were put into the scrotum without any difficulty. A subdartos pouch was created in all patients, and the orchiopexy was completed. Scrotal edema and wound infection occurred in five and two patients, respectively, in the first postoperative week.

The mean follow-up period was 2.5 years (range 8 months to 9 years). Testicular atrophy occurred in one patient, and recurrent UT occurred in three. One of these three patients underwent correction by the Prentiss maneuver, and the remaining two underwent classical orchiopexy. No patients developed an inguinal hernia. All patients were treated by pediatric surgeons on an outpatient basis.

## DISCUSSION

At about the time of birth, the portion of the PV between the testicle and the abdominal cavity is obliterated, leaving the peritoneal cavity separate from the PV, which surrounds the testicle. If obliteration of the PV is incomplete, a variety of anomalies can occur. Most true cases of UT (90%) are associated with a PPV, with the exception of retractile testes.

In one series, laparoscopy was performed in patients with UT in whom the testes were non-palpable; the testes were visualized in 47 patients, and 43 (91%) had a PPV. Of 77 patients in whom the PV was closed, 75 (97%) had vanished or absent testes.^6^ In another study, epididymal anomalies in patients with PPV included inguinal hernia, hydrocele, and UT. The incidence of PPV associated with UT was higher than that of other inguinal anomalies (69%), and epididymal anomalies were more frequently associated with undescended (72%) than descended (34%) testes. The study concluded that androgenic stimulation is mandatory for PV closure and that PPV occurs with a high incidence in patients with UT.^7^ In another study by Radmayr et al.,^8^ inguinal hernias were detected in 26% of patients with UT.

Traditionally, the recommended treatment for UT is high ligation of the hernial sac after proper dissection up to the deep ring. Jain et al.^4^ evaluated 450 cases of orchiopexy in male patients from 6 months to 10 years of age. None of them had demonstrable hernias. All of the children underwent the orchiopexy san ligation technique of orchiopexy. The sac was opened directly while keeping the testis down and separated from the cord structures up to the deep ring. In their technique, the sac remained unligated and was pushed below the deep ring. No patients developed inguinal hernias in the follow-up period. However, they selected patients in whom the testes were palpable.^4^ Mohta et al. suggested not ligating the sac in pediatric inguinal hernia repair.^5^ A prospective study of laparoscopic inguinal hernia repair in children by Schier^9^ showed no difference between simple suturing and resection of the hernia sac. In addition, it was emphasized that an open internal inguinal ring is not an inguinal hernia. During laparoscopic orchiopexy, Handa et al.^10^ showed that closure of the internal ring is not necessary. Blackburn et al.^11^ incised the indirect hernia sac but did not perform high ligation in their case series of Fowler–Stephens orchiopexy. On the other hand, Varela-Cives et al.^12^ investigated PPV in patients with UT who underwent herniography. Ninety-five of 376 patients with UT had PPV (25.3%), and 31 of 244 patients without UT had PPV (12.7%); the difference between these two groups of patients reached statistical significance (*P*=0.0001). The incidence of PPV was higher at younger ages and reached 41.3% among all patients with NPT.^12^ In two other prospective studies of pediatric patients with UT (excluding those with clinically overt hernia and hydrocele), surgical exploration revealed a PPV prevalence of 71% and 77%, respectively.^13^,^14^ In patients with UT, the presence of PPV is related to both patient age and testis position. In patients aged <2 years, the prevalence of PPV was 68.7%. This percentage was dramatically lower at 27.2% in patients aged 2–6 years and lower still at 11.2% in patients aged 6–12 years.^12^ Undescended testis located cranial to the external inguinal ring was significantly more frequently associated with PPV than UT located more caudally.

In concordance with the literature, we perform PV dissection in all of our patients with UT to aid the performance of the orchiopexy. Patients with NPT were specifically chosen for the present study because the incidence of PPV is higher in such cases. During the orchiopexy procedure in these patients, the PV should be dissected and high ligation should be performed to provide a tension-free repair. If an overt hernia is present, expeditious hernia repair with orchiopexy at the age of presentation is undertaken. Otherwise, the hernia should be repaired at the time of orchiopexy. Riquelme et al.^15^ found inguinal hernias in 23 cases (69.9%): in 16 (76.%) palpable testes and 7 (58.3%) NPT during laparoscopic orchiopexy of 64 patients. They found no inguinal hernias on the side opposite the UT.^15^ In our study, 7 patients were suspected to have a hernia by the family, and 22 patients with the silk sign were diagnosed by physical examination during a visit to a physician. A wide PPV was observed intraoperatively in all patients with NPT.

We observed no hernias in the follow-up period after PV dissection with high ligation, suggesting the suitability of this procedure. We chose to perform open orchiopexy because we believe that thorough dissection and very high ligation are key factors in the success of the procedure. Furthermore, this approach allows for controlled incision of the PV and access to the abdomen to search for the UT. Patent PV dissection is initiated between the internal ring and testis. This enables dissection of a smaller circumference of the PV and enables better repair if the PV is inadvertently ruptured than does repair from the inner ring level. Very high ligation of the PV enables a tension-free orchiopexy outcome. Furthermore, it supports performance of the Prentiss maneuver and prevents hernia formation in patients with a wide PPV.

## CONCLUSION

During open or laparoscopic orchiopexy, especially in patients with UT involving NPT, the PV should be dissected and high ligation should be performed to prevent hernia formation and provide a good outcome.

## Abbreviations

NPT: non-palpable testis
PPV: patent processus vaginalis
PV: processus vaginalis
UT: undescended testis

## References

[1] Berkowitz GS, Lapinski RH, Dolgin SE, Gazella CA, Holzman IR (1993). Prevalence and natural history of cryptorchidism. Pediatrics.

[2] Levitt SB, Kogan SJ, Engel RM (1978). The impalpable testis: a rational approach to management. J Urol.

[3] Leung AK, Robson WL (2004). Current status of cryptorchidism. Adv Pediatr.

[4] Jain VK, Singh S, Garge S, Joshi M, Sanghvi J (2011). Orchidopexy san ligation technique of orchidopexy. Afr J Paediatr Surg.

[5] Mohta A, Jain N, Irniraya KP, Saluja SS, Sharma S, Gupta A (2003). Nonligation of hernial sac during herniotomy: a prospective study. Pediatr Surg Int.

[6] Elder JS (1994). Laparoscopy for impalpable testes: significance of the patent processus vaginalis. J Urol.

[7] Barthold JS, Redman JF (1996). Association of epididymal anomalies with patent processus vaginalis in hernia, hydrocele and cryptorchidism. J Urol.

[8] Radmayr C, Corvin S, Studen M, Bartsch G, Janetschek G (1999). Cryptorchidism, open processus vaginalis, and associated hernia: laparoscopic approach to the internal inguinal ring. Eur Urol.

[9] Schier F (2006). Laparoscopic inguinal hernia repair – a prospective personal series of 542 children. J Pediatr Surg.

[10] Handa R, Kale R, Harjai MM (2005). Laparoscopic orchiopexy: is closure of the internal ring necessary?. J Postgrad Med.

[11] Blackburn SC, Adams SD, Mahomed AA (2012). Risk of hernia occurrence where division of an indirect inguinal sac without ligation is undertaken. J Laparoendosc Adv Surg Tech A.

[12] Varela-Cives R, Bautista-Casasnovas A, Taboada-Santomil P (2008). Relevance of herniography for accurate diagnosis of patent processus vaginalis in cryptorchidism. Int Braz J Urol.

[13] Adamsen S, Aronson S, Borjesson B (1989). Prospective evaluation of human chorionic gonadotropin in the treatment of cryptorchidism. Acta Chir Scand.

[14] Hazebroek FW, de Muinck Keizer-Schrama SM, van Maarschalkerweerd M, Visser HK, Molenaar JC (1987). Why luteinizing-hormone-releasing-hormone nasal spray will not replace orchiopexy in the treatment of boys with undescended testes. J Pediatr Surg.

[15] Riquelme M, Aranda A, Rodriguez C, Cortinas J, Carmona G, Riquelme-Q M (2007). Incidence and management of the inguinal hernia during laparoscopic orchiopexy in palpable cryptoorchidism: preliminary report. Pediatr Surg Int.

